# Glia and semaphorins in neurodegenerative diseases: The frontier for new therapeutics

**DOI:** 10.4103/NRR.NRR-D-25-00223

**Published:** 2025-06-19

**Authors:** Sofia Nutarelli, Claudia Palazzo, Maria Teresa Viscomi

**Affiliations:** Department of Life Science and Public Health Section of Histology and Embryology, Università Cattolica del Sacro Cuore, Rome, Italy; Fondazione Policlinico Universitario “A. Gemelli”, IRCCS, Rome, Italy

The optimal development, function, and maintenance of the central nervous system (CNS) are determined by the dynamic and continuous crosstalk between its components. Neurons and glial cells, the cellular constituents of the CNS, orchestrate a wide range of essential activities (Allen and Lyons, 2018). Notably, glial cells, which outnumber neurons, constitute the major population within the CNS. This population comprises astrocytes, microglia, oligodendrocytes, and ependymal cells, each fulfilling specialized functions that contribute to neural homeostasis and overall CNS integrity. Astrocytes are pivotal in preserving structural and functional integrity through the regulation of synaptic function, the clearance of neurotransmitters, and ion balance. Moreover, they provide metabolic support to neurons. Microglia are resident immune cells that provide continuous surveillance within the CNS, regulating brain development and maintenance of neuronal networks. Oligodendrocytes are responsible for myelination, thereby modulating the speed of action potential conduction and optimizing neural communication. Lastly, ependymal cells, which form the epithelial lining of the brain’s ventricular system, are essential for the production of cerebrospinal fluid, its regulation, and the clearance of waste, thus playing a pivotal role in brain metabolism.

Glial cells differ from neurons in morphology, signaling mechanisms, and the spatial and temporal scales at which they function. Glial cells are electrically non-excitable, a characteristic that contributed to their historical underrepresentation in neuroscientific research, where their study was initially confined to morphological observations. However, despite these differences, glial cells and neurons share a common neuroectodermal origin and utilize neurotransmitter-based signaling systems, underscoring their functional interplay in CNS physiology.

Several state-of-the-art technologies such as single-cell RNA sequencing and single-nucleus RNA sequencing are making considerable contributions to increase our understanding on the complexity of glial cells, and in particular, unveiling the molecular mechanisms that allow glia responses to a wide variety of environmental challenges.

As a result of these advances, glial biology, which has long focused on the functions of individual glial subtypes, has recently revealed that glial cells engage in many interactions not only with neurons but also among themselves (Lopez-Ortiz and Eyo, 2024).

In the mammalian CNS, communication between neurons and glia is primarily facilitated by neurotransmitters. Glial cells also use other molecules such as cytokines, chemokines, adenosine triphosphate, and growth factors to communicate with their counterparts. In recent years, the repertoire of these molecules has expanded significantly, with the inclusion of additional factors such as semaphorins (for a comprehensive review, see Palazzo et al., 2025).

In the CNS, semaphorins and their related receptors, plexins, have been shown to play a pivotal role in a multitude of processes, including neurogenesis, wiring, and cell migration during development (Limoni and Niquille, 2021). Beyond these functions, semaphorins have been shown to regulate diverse forms of cell‒cell communication in developmental, physiological, and pathological contexts (Carulli et al., 2021). Semaphorins are categorized into eight distinct classes based on sequence similarity and domain structure. Invertebrate semaphorins are predominantly classified into classes 1 and 2, whereas classes 3 to 7 are observed in vertebrates, and class 5 encompasses semaphorins encoded by viral genomes. A salient feature of semaphorins is the highly conserved extracellular N-terminal domain, known as the Sema domain, which is instrumental for dimerization and receptor binding. Semaphorins belonging to classes 4, 5, and 6 are membrane-bound, while those in classes 2 and 3 are secreted and class 7 comprises GPI-anchored members. The transmembrane semaphorins function as bidirectional signaling molecules, acting as ligands for their receptor plexins (forward signaling) or as receptors themselves, initiating reverse signaling through their intracellular tail (Battistini and Tamagnone, 2016).

Semaphorins, traditionally studied for their roles in axonal guidance, have gained recognition for their dynamic roles in modulating glial interactions, thanks to the binding with their cognate receptors. These pathways represent a transformative frontier in the quest for innovative CNS therapies, particularly for neurodegenerative disorders. Despite the considerable heterogeneity exhibited by these diseases with regard to their underlying pathogenetic mechanisms, it is notable that they are all unified by the hallmark feature of neurodegeneration, which is associated with neuroinflammation.

In recent years, the work of Clark et al. (2021), one of the pioneering studies in the field, has provided a sophisticated illustration of semaphorins as mediators of microglia-astrocyte crosstalk that can drive CNS pathology. This was achieved through the development of rabies barcode interaction detection followed by sequencing, a new tool for the high-throughput identification of cell-cell interactions and molecular characterization of interacting cells at single-cell resolution. Notably, the study identified semaphorin 4D (Sema4D) as a potential mediator in glia-glia interactions that govern disease advancement in an experimental autoimmune encephalomyelitis model of inflammatory demyelinating disease that mimics multiple sclerosis (MS). The investigation of ligand-receptor interactions between microglia and astrocytes proved instrumental in accentuating the preferential interaction between Sema4D-positive microglia and PlexinB2-positive astrocytes. This finding provides a novel perspective on their contribution to the inflammatory context of MS. The robustness of this finding was further corroborated in MS patients, where a published single-cell RNA sequencing dataset of MS patients and matched controls showed increased interactions between Sema4D-positive microglia and PlexinB2-positive astrocytes (Clark et al., 2021). Specifically, the authors reported that in MS patients, the presence of a pro-inflammatory signature was associated with PlexinB2-positive astrocytes interacting with Sema4D-positive microglia. More interestingly, in another preclinical model of MS, the cuprizone mouse model, which recapitulates several aspects of progressive MS pathology, Enrich-Bengoa et al. (2022) identified Sema4D-PlexinB1 as a key mediator of microglia‒oligodendrocyte progenitor cell interactions during the demyelinating phase characterized by oligodendrocytes degeneration and activation of the innate immune system.

In a similar vein, recent studies utilizing an experimental model of Alzheimer’s disease have elucidated the role of PlexinB1 signaling in mediating astrocyte-microglia communication in the context of neurodegeneration mediated by neuroinflammation. In Alzheimer’s disease, which is characterized by extracellular amyloid plaques and intracellular neurofibrillary tangles surrounded by reactive microglia and astrocytes, PlexinB1 has been identified as a differentially regulated gene in late-onset Alzheimer’s disease, particularly upregulated in plaque-associated astrocytes. Specifically, PlexinB1 expression in reactive peri-plaque astrocytes was found to negatively regulate microglia recruitment, enforcing cell spacing and limiting glial coverage of amyloid-beta deposits, leading to more diffuse amyloid plaques and greater neuronal damage. Depletion of PlexinB1 reduced astrocyte and microglia reactivity, increased microglial coverage of amyloid-beta deposits, and, concomitantly, reduced neuroinflammation (Huang et al., 2024).

Similarly, more recently, in an acute mouse model of brain disease characterized by cortical injury, astrocyte‒microglia interaction was found to be mediated by Sema4B coupled to PlexinB2 (Casden et al., 2024). The occurrence of brain injury instigates an immediate response aimed at restoring homeostasis and limiting damage. A concerted activation of microglia and astrocytes is a critical part of this response. Following cortical injury, Sema4B expression in cortical astrocytes was able to enhance the reactivity of microglia via PlexinB2, thereby increasing the expression of several inflammatory cytokines and leading to neuronal death.

**Figure 1:**
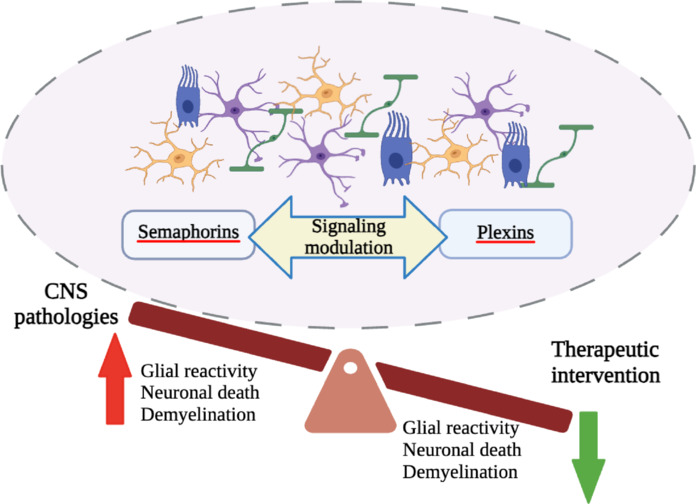
Schematic of glial cell crosstalk mediated by semaphorin/plexin signaling in neurodegenerative diseases. In recent years, semaphorins have been identified as critical mediators of glia–glia interactions in several central nervous system (CNS) pathologies, such as multiple sclerosis and Alzheimer’s disease (see the text). Glial cells such as astrocytes (purple), microglia (orange), oligodendrocytes (green), and ependymal cells (blue) are involved in neuronal support and protection, synaptic transmission, and CNS homeostasis. Indeed, glia–glia miscommunication has been shown to contribute to the development and progression of several CNS pathologies. When CNS homeostasis is disturbed, glial cells, particularly astrocytes and microglia, initiate a multimodal inflammatory response aimed at restoring balance. In several CNS pathologies, the semaphorin-plexin signaling pathway has been implicated in neuroinflammatory responses through its involvement in glia-glia communication. Upregulation of this pathway results in increased glial cell reactivity, leading to neuronal death and, in multiple sclerosis, to axonal demyelination (as indicated by the red arrow). Given the central role of glial cells in the initiation and progression of many neurological diseases and their function as important mediators of neuroinflammation, modulation of semaphorin signaling in this area has the potential to pave the way for the development of new therapeutic interventions. Therapeutic interventions aimed at modulating semaphorins mediated by glia-glia interactions in pathological settings have the potential to reduce neuroinflammation-associated neurodegeneration and restore CNS homeostasis (as highlighted by the green arrow). The figure summarizes the studies that have been examined in the text. Created with BioRender.com.

Collectively, these emerging studies highlight the pivotal role of semaphorins as key regulators of glial communication and responses, particularly in the context of altered CNS homeostasis (**Figure 1**). Nevertheless, the intricacies of semaphorin signaling in glia-glia communication in different disease contexts remain to be fully elucidated. This complexity arises from the multifaceted nature of the glial cells implicated, as well as the intricate signaling pathways that operate within the inflamed brain regions. These are merely a few of the numerous issues that require resolution. Furthermore, the observation that the process of neuroinflammation is initiated by a local response of glial cells to alterations in the microenvironment underscores the need for further research to elucidate the role of semaphorin signaling in other pathways traditionally implicated in this mechanism, including nuclear factor κB, p38 mitogen-activated protein kinase, and Akt/mammalian target of rapamycin. However, given that semaphorins have only recently been identified as potential targets for modulating glia-glia interactions in CNS pathologies, these and many other aspects need to be considered in the formulation of future semaphorin-targeted therapeutic interventions. To gain a holistic understanding of how semaphorins may contribute to CNS diseases, crucial for the design of therapeutic strategies, future studies need to address multiple aspects. These should include the pathological aspects of diseases induced by altered glial reactivity, the functional players in semaphorin signaling and their synergistic effects.

In recent years, increasing evidence has demonstrated the pivotal role of aberrant interactions between cells, inflammatory mediators, and signaling pathways in the abnormal activation of the immune system that characterizes neurodegenerative disorders. This abnormal activation not only contributes to their progression, but also serves as a major catalyst in its initiation. In this inflammatory context, the heterogeneity of the different glial populations at different stages of the disease or in different brain regions underlines the possibility of multiple cellular functions during the pathology. This could also be assumed for semaphorin-plexins, which may act as a double-edged sword, exerting a protective and/or damaging effect at different stages of the disease process. Further studies are needed to elucidate the contribution of semaphorin-plexin signaling to glial cells crosstalk during the different diseases. However, there is currently a lack of research in this area. Even under physiological conditions, there is limited evidence for their role in different life stages and different brain regions (reviewed in Palazzo et al., 2025). Advances in single-cell and spatial transcriptomics hold the potential to facilitate precise temporal and spatial mapping of glia-glia interactions and in the expression profiles of semaphorins and plexins in the different regions of the CNS in different diseases. This, in turn, may allow future development of selective modulation of semaphorins/plexins in specific glial subpopulations at specific times in the disease process, thereby minimizing off-target effects. This may be advantageous given the multiple functions of glial cells. Indeed, therapeutic interventions targeting these pathways hold great promise for the treatment of neuroinflammation-mediated neurodegeneration, as they allow modulation of the balance between pro-inflammatory and protective responses in the nervous system. Furthermore, given the direct and/or indirect involvement of semaphorin-plexin signaling in neuronal dysfunction via glial cells in several diseases, treatments that block potentially pathogenic semaphorin signaling could alleviate disease-related symptoms and improve patients’ quality of life.

In the future elaboration of therapeutic approaches, it is also imperative to recognize the importance of several functional components within semaphorin-plexin signaling pathways, including their capacity for bidirectional signaling. Transmembrane semaphorins have been shown to act as both ligands (forward signaling) and receptors (reverse signaling). This dual role opens up opportunities for more nuanced therapeutic strategies, where interventions could simultaneously target forward and reverse signaling to restore healthy glial communication. Furthermore, it is important to consider that in certain pathological contexts (e.g., demyelinating diseases), the presence of a single ligand (i.e., Sema4D) in microglia, but multiple receptors (e.g., PlexinB2 and PlexinB1) expressed by different glial cell types (e.g., astrocytes and oligodendrocytes) may lead to functional receptor redundancy, which may be critical in the inflammatory setting. In this last scenario, to effectively design a therapeutic plan, it is necessary to identify the individual and cumulative functions of the receptors with respect to their role in the inflammatory context.

In addition to their therapeutic potential, semaphorins and plexins may also have utility as biomarkers for CNS diseases. Elevated levels of certain semaphorins, such as Sema4D, have been observed to correlate with disease severity and progression in MS. Consequently, the measurement of Sema4D expression or activity could enhance the accuracy of early diagnosis, facilitate the monitoring of disease progression, and predict therapeutic response.

In view of the findings outlined, the semaphorin-plexin axis may provide a valuable source of inspiration for the future design of therapeutic strategies. As our understanding of these pathways deepens, we can envisage therapies that could: block maladaptive semaphorin-plexin signaling in acute and chronic neuroinflammation; enhance beneficial interactions to support neuronal survival and CNS repair; and/or combine semaphorin-plexin modulation with other therapies for synergistic effects. By targeting this central communication hub, it may be possible to develop powerful strategies to halt the progression of neurodegeneration and restore functions. The advent of this novel approach may signify the commencement of a new era characterized by advancements in precision medicine for glial biology.

*This work was supported by Linea D.1. 2023-24 Università Cattolica del S. Cuore (to MTV).*
